# Augmenting project ECHO for opioid use disorder with data-informed quality improvement

**DOI:** 10.1186/s13722-023-00381-2

**Published:** 2023-04-28

**Authors:** Owen B. Murray, Marcy Doyle, Bethany M. McLeman, Lisa A. Marsch, Elizabeth C. Saunders, Katherine M. Cox, Delitha Watts, Jeanne Ryer

**Affiliations:** 1 Northeast Node of the Clinical Trials Network, Center for Technology and Behavioral Health, Geisel School of Medicine at Dartmouth College, 46 Centerra Parkway, Suite 315, NH 03766 Lebanon, USA; 2 New Hampshire Citizen’s Health Initiative, Institute for Health Policy and Practice, University of New Hampshire, 2 White Street, NH 03301 Concord, USA

**Keywords:** Primary care, Medications for opioid use disorder, Quality improvement, Buprenorphine, ECHO

## Abstract

**Background:**

National opioid-related overdose fatalities totaled 650,000 from 1999 to 2021. Some of the highest rates occurred in New Hampshire, where 40% of the population lives rurally. Medications for opioid use disorder (MOUD; methadone, buprenorphine, and naltrexone) have demonstrated effectiveness in reducing opioid overdose and mortality. Methadone access barriers disproportionally impact rural areas and naltrexone uptake has been limited. Buprenorphine availability has increased and relaxed regulations reduces barriers in general medical settings common in rural areas. Barriers to prescribing buprenorphine include lack of confidence, inadequate training, and lack of access to experts. To address these barriers, learning collaboratives have trained clinics on best-practice performance data collection to inform quality improvement (QI). This project sought to explore the feasibility of training clinics to collect performance data and initiate QI alongside clinics’ participation in a Project ECHO virtual collaborative for buprenorphine providers.

**Methods:**

Eighteen New Hampshire clinics participating in a Project ECHO were offered a supplemental project exploring the feasibility of performance data collection to inform QI targeting increased alignment with best practice. Feasibility was assessed descriptively, through each clinic’s participation in training sessions, data collection, and QI initiatives. An end-of-project survey was conducted to understand clinic staff perceptions of how useful and acceptable they found the program.

**Results:**

Five of the eighteen health care clinics that participated in the Project ECHO joined the training project, four of which served rural communities in New Hampshire. All five clinics met the criteria for engagement, as each clinic attended at least one training session, submitted at least one month of performance data, and completed at least one QI initiative. Survey results showed that while clinic staff perceived the training and data collection to be useful, there were several barriers to collecting the data, including lack of staff time, and difficulty standardizing documentation within the clinic electronic health record.

**Conclusions:**

Results suggest that training clinics to monitor their performance and base QI initiatives on data has potential to impact clinical best practice. While data collection was inconsistent, clinics completed several data-informed QI initiatives, indicating that smaller scale data collection might be more attainable.

## Background

An estimated 650,000 opioid-related overdose fatalities occurred nationally from 1999 to 2021 [1, 2]. New Hampshire, where 40% of the population lives in rural areas [3], ranked consistently among states with the highest rate of opioid-involved overdose deaths per capita (second from 2014 to 2016 and fourth from 2017 to 2018) [4, 5]. Medications for opioid use disorder (MOUD), including methadone, naltrexone, and buprenorphine [6], have demonstrated effectiveness in reducing opioid overdose and mortality [7, 8]. However, people seeking MOUD in rural areas have reported facing barriers such as traveling greater distances to access treatment and having fewer available providers [9–11]. Access to methadone for Opioid Use Disorder (OUD) can be particularly challenging for patients, particularly in rural regions [12, 13], and extended-release naltrexone trials have found limited patient uptake and frequent premature cessation[14, 15]. In contrast, treatment with buprenorphine has expanded access to MOUD [16]. The number of buprenorphine prescriptions written in primary care offices nationally increased substantially from 2006 to 2019 [16–18], although availability may still be insufficient to meet need for care [19].

Successive regulatory changes to buprenorphine prescription guidelines have enhanced potential MOUD access, of particular relevance to rural areas where methadone for OUD and prescribing physicians are scarcer [10–13]. In 2017, federal regulation changes allowed nurse practitioners and physician assistants to prescribe buprenorphine [20, 21] and in 2021, federal training requirements for buprenorphine waivers were exempted in for prescribers treating up to 30 patients [22]. However, many waivered prescribers have not offered buprenorphine at or near their regulatory capacity [23, 24] suggesting that even the elimination of the buprenorphine waiver requirement as of December 29, 2022 [25, 26] may not entirely alleviate access barriers. Providers have cited numerous barriers to prescribing buprenorphine including a lack of confidence, inadequate staff training, and lack of access to experts [27–29].

Models to address these barriers to expanding buprenorphine prescribing have been studied and implemented in various settings and situations. One such model, Project ECHO® (Extension for Community Healthcare Outcomes), is an evidence-based method using web-based teleconferencing linking subject matters experts with community-based sites assisting health care teams in treating complex illnesses [30, 31]. Project ECHO was designed to support rural primary care providers; the initial iteration supported primary care in rural New Mexico in treatment of Hepatitis C without the need to refer patients to distant specialty providers [30]. Project ECHO applied to OUD care with buprenorphine [31, 32] has been shown to improve provider knowledge and self-efficacy [33, 34]. The Best-practice in Oral Opioid agoniSt Therapy (BOOST) Collaborative model employed in Vancouver, Canada utilized quality improvement (QI) coaching for MOUD with buprenorphine, methadone, or slow release oral morphine [35]. This urban initiative involved monthly in-clinic visits by a support team consisting of a medical lead, a Collaborative lead, and a QI coach who supported development of quality indicator reports derived from clinic EHR data and facilitated clinic-identified QI initiatives [35]. The BOOST Collaborative QI coaching model was associated with improved retention in OUD care, improved OUD care processes, and improved quality of SUD care [35]. Clinic-level coaching for addiction services providers has also been associated with increased rates of patients treated for substance use disorders (SUDs) and reduced wait times for patients to access SUD treatment [36].

A care model in the rural state of Vermont, New Hampshire’s neighbor, is the Vermont Hub and Spoke System (HSS). This model was a statewide expansion of MOUD delivered by a network of office-based community buprenorphine providers (spokes) and more intensive specialized centers providing methadone or buprenorphine (hubs) [37–39]. In the HSS, Medicaid funding supports spokes with addiction-specialist nurses and behavioral health clinicians [37–39]. After HSS implementation, Vermont contracted a team at Dartmouth College on a QI project that combined a best practice didactic and case-based learning collaborative with facilitated clinical performance tracking [40] and Plan-Do-Study-Act (PDSA) rapid-cycle QI initiatives [41]. PDSA tools have been utilized for QI during buprenorphine for OUD learning collaboratives [35, 40] and buprenorphine telehealth transition [42]. Systematic collection, aggregation, and reporting of performance data during learning collaborative sessions contributed to the impact of Vermont’s HSS. The learning collaborative saw 85.7% engagement from spoke physicians, statistically significant improvements on more than half of all performance data metrics collected, and a decrease in practice variation on all measures over time [40]. Since implementation of the HSS, MOUD access increased dramatically in Vermont, and by 2017 the state had the nation’s highest per capita capacity to provide buprenorphine [37, 38] and high levels of buprenorphine distribution to pharmacies relative to other states [43].

New Hampshire, a state ravaged by the opioid epidemic [4], worked to increase MOUD capacity by launching several programs to increase access to buprenorphine [44, 45]. In April 2019, the New Hampshire Citizens Health Initiative at the University of New Hampshire launched the Partnership for Academic-Clinical Telepractice: Medications for Addiction Treatment (PACT-MAT) Project ECHO to provide didactic content and collaborative review of peer-presented cases to New Hampshire buprenorphine providers. With the PACT-MAT ECHO serving as the learning collaborative, this training study, the ECHO-Augmented Medications for Addiction Treatment Practice Learning to Implement Facilitated Quality Improvement (ECHO-AMPLIFI; funded by the National Institute on Drug Abuse, National Drug Abuse Treatment Clinical Trials Network [(CTN) Protocol 0103] introduced performance data-driven QI facilitation to participating practices. This study sought to explore the feasibility of implementing performance-based data collection to inform PACT-MAT ECHO participant QI initiatives to increase provider knowledge, awareness, and comfort related to best practices for buprenorphine patient management such as SUD and OUD diagnosis, treatment engagement and retention, periodic toxicology testing and Prescription Drug Monitoring Program (PDMP) review, and access to naloxone for overdose reversal [46–48].

## Methods

### Project design

ECHO-AMPLIFI was developed as an additional component of the PACT-MAT ECHO to explore the feasibility of training clinic staff to collect and review performance data and initiate QI initiatives to achieve best practice of OUD care with buprenorphine. Participating clinics were given data collection tools, including HIPAA-compliant Excel spreadsheets in which to enter nonidentifying patient-level and clinic-level performance data, pre-formulated tables and figures to explore those data over time, and a workbook that detailed the best practice related to each metric. Performance measures were developed based on best practices covered by the PACT-MAT ECHO sessions. The ECHO-AMPLIFI team trained participating clinic staff in how to collect performance metric data, how to review those data during team meetings, and how to initiate rapid-cycle QI initiatives to direct change on a given metric. ECHO-AMPLIFI was intended to run concurrently with the PACT-MAT ECHO (April-December, 2019) but ran from July 2019 to March 2020. Experienced practice facilitators from the New Hampshire Citizens Health Initiative (NHCHI) joined a project team from Dartmouth College involved with the original Vermont HSS learning collaborative. The Dartmouth team worked with clinics to elucidate the rationale behind each best practice, help clinic staff collect their performance data, and assist in review of performance metric results to identify targets for rapid cycle QI initiative. NHCHI practice facilitators worked with clinic teams throughout this process to support clinic QI initiatives.

Clinic trainings were scheduled in advance for the six months that the PACT-MAT ECHO and ECHO-AMPLIFI ran concurrently. Three additional sessions were offered from January to March, 2020 for a potential total of nine sessions with ECHO-AMPLIFI practice facilitators and data collection experts. Initial training sessions on data entry and chart audit procedures were held at each clinic to tailor data collection procedures to each clinic’s electronic health record (EHR) system. The project team continued to meet with each clinic monthly to review the collected performance data and assist clinics in implementing rapid-cycle QI initiatives using the PSDA tool [41] and Process Mapping, a graphic representation of targeted clinical procedures [49]. Discussions about performance data, in-progress data collection, barriers to data collection, and QI initiatives related to the measures were documented by the practice facilitators after monthly discussion sessions in Quality Improvement Workbooks. Updates to the Quality Improvement Workbooks were uploaded to clinic cloud storage accounts provided by the project or emailed to clinic staff.

In addition, three video-conference sessions and unlimited remote support between sessions were provided. The first videoconference was an introduction to project goals and procedures, the second provided cross-clinic sharing of project implementation, and the final videoconference offered a forum to share observations about ECHO-AMPLIFI project participation (Table 1).

**Table 1:** ECHO-AMPLIFI program activities

**Timeline**	July 17, 2019	July 2019 -March − 2020	October 16, 2019	February 12, 2020	February 12 and 18, 2020	March 9, 2020
**Activity**	Introductory video-conference	Monthly QI facilitation	Conjoint implementation video-conference	Final video-conference	ECHO-AMPLIFI Survey distributed	Activities completed

### ECHO-AMPLIFI performance measures

Performance data collection procedures were designed to complement learning from the PACT-MAT Project ECHO modules and inform QI cycles. Performance data measures similar to the Vermont HSS learning collaborative measures [40] were created to reinforce PACT-MAT Project ECHO sessions on naloxone overdose reversal device access, practice workflow, and behavioral aspects of OUD management such as screening, buprenorphine initiation, and retention (Table 2). Each measure tracked clinical performance regarding a best practice recommendation published by federal and state agencies [46, 47, 50] or by the American Society of Addiction Medicine [48]. A Toolkit was developed to elucidate the rationale for each measure and specify how to collect and review data to inform QI initiatives identified by the clinic. Clinics were encouraged to submit data monthly while participating in the PACT-MAT Project ECHO. As this was a feasibility study to examine training clinics to conduct data collection to inform QI initiatives, these performance measures were not analyzed, and data are not reported herein.

**Table 2:** ECHO-AMPLIFI performance data measures

Outcome	Numerator/denominator	Description
**Practice procedures: treatment initiation**		
1. Number of days from contact to prescription		Number of days from first request for MAT from patient or referring agency to date of first prescription
2. Number of days between assessment and prescription		Number of days between documented MAT assessment/diagnostic criteria and date of first prescription (potentially a negative number)
3. Percent of clinic patients screened for substance use	Number of patients completed screening/Patients checked in for screening eligible clinic visits	Routine screening performance per practice using an evidence-based tool. Collect number of patients in the month who checked in for screening eligible clinic visits and number of patients screened for SUD
4. Percent of patients with OUD diagnostic criteria documented at assessment	Number with diagnostic criteria documented/Number of all patients on MAT	Assurance that medications are being prescribed to people who meet diagnostic criteria for OUD. Collect number of MAT patients with diagnosis documented using ICD-10 or DSM-5 criteria
5. Percent of patients on MAT for whom the PDMP was queried before first prescription	Number of patients with initial PDMP query/Number of all patients on MAT	Ascertain if PDMP was queried on date of first prescription or in the preceding month
**Practice procedures: ongoing care**		
6. Average length of current MAT episode of care	Number of days in active MAT/7 (days calculated by subtracting date of first prescription for this episode of care from date of most recent prescription. Number of days then averaged for total number of active MAT patients)	Practice-level average number of weeks MAT patients are in treatment Collect date of first MAT prescription from this episode of care and date of most recent MAT prescription
7. Percent of patients on MAT for whom naloxone has been provided or prescribed	Number prescribed naloxone or provided a kit/Number of all patients on MAT	Collect number of MAT patients prescribed or provided naloxone by the practice
8. Percent of patients on MAT for whom the PDMP was queried every 3 months	Number of patients with PDMP query every 3 months/Number of all patients on MAT	Collect date of most recent PDMP query
9. Percent of patients on MAT receiving drug tests at least monthly	Number of patients who received drug tests monthly/Number of all patients on MAT	Collect date of most recent drug test
10. Percent of patients diagnosed with OUD not receiving MAT	Number prescribed MAT + number receiving MAT elsewhere /Number with OUD diagnosis in chart	MAT is the standard of care for patients with OUD. Collect the number of current MAT patients and all patients at the practice with an OUD diagnosis.

### Outcome measures

Feasibility of implementing performance data informed QI initiatives among clinics also participating in the PACT-MAT ECHO was measured through an examination of each clinic’s engagement in ECHO-AMPLIFI training sessions, data collection, QI initiatives, and subjective feedback provided via a survey of participating clinic staff. Engagement was determined by clinics attending at least one session and videoconference, making at least one request for remote support, completing at least one data submission, and identifying at least one QI initiative for the clinic to work on. Upon completion of project activities, the link to an anonymous web-based survey consisting of nine Likert scale items and four open-ended response items was distributed to all participating clinic staff (n = 18). One reminder was emailed to those who did not complete the survey during the first week. The survey was developed for the ECHO-AMPLIFI study and assessed perceptions of relevance and usefulness of data collection processes and QI facilitation, perceived impact on MOUD service delivery, and perceived relationship of ECHO-AMPLIFI participation with PACT-MAT Project ECHO.

### Recruitment

Clinics were recruited from the active PACT-MAT Project ECHO that began in April 2019. All clinics participating in the PACT-MAT Project ECHO were sent flyers inviting participation in ECHO-AMPLIFI and were incentivized by a $1,500 dollar payment for data collection efforts. Of the 18 clinics participating in the ECHO, a convenience sample of five clinics agreed to participate in ECHO-AMPLIFI. The remaining 13 clinics did not respond or declined ECHO-AMPLIFI participation.

### Analysis

The primary outcome of this study was to explore the feasibility of training New Hampshire clinics engaged in a Project ECHO for OUD management with buprenorphine to collect data on their performance on best practice metrics and to engage in QI initiatives based on the data. Feasibility was measured via assessing clinics’ engagement in monthly ECHO-AMPLIFI sessions, videoconferences, remote support requests, performance data submissions, QI initiatives, and an end-user survey. Primary outcome analysis of feasibility was descriptive. Characteristics of the participating clinics and survey results were calculated using means and standard deviation or number and percentages, as appropriate.

## Results

### Site characteristics

Five of 18 eligible clinics participated in ECHO-AMPLIFI, a 28% enrollment rate. Four of the five clinics were in rural towns in New Hampshire (80.0%) with an average of 32,000 inhabitants (range: 2000–112,000) between the five areas. Two of the clinics were affiliated with larger hospitals, two were independent primary care clinics including a Federally Qualified Health Center (FQHC) and an FQHC look-alike (meeting federal Health Resources and Services Administration requirements without receiving funding), and one was a community mental health center. Site staff involved in ECHO-AMPLIFI ranged from clinic leadership to buprenorphine prescribers to social workers. All five participating clinics were treating patients with buprenorphine when they joined the project. Four of the five clinics were treating fewer than ten patients with buprenorphine when the project began in July of 2019. The five clinics were collectively treating 59 patients with buprenorphine when they began ECHO-AMPLIFI activities and 106 patients at project end (Table 3).

**Table 3:** Characteristics of clinics participating in the ECHO-AMPLIFI project (n = 5)

	Clinic 1	Clinic 2	Clinic 3	Clinic 4	Clinic 5	All clinics
**Clinic description**	Community Mental Health	FQHC^a^	FQHC Look-Alike±	Hospital-Affiliated Primary Care A	Hospital-Affiliated Primary Care B	
**Clinic team leaders**	Quality assurance and service development directors	Waivered buprenorphine prescriber	Social worker	Clinical Operations Director	Waivered buprenorphine prescriber	
**Clinic team composition**	Behavioral health clinicians, quality assurance, data analyst	Physicians and population health specialist	Infrequent participation by other staff	Quality assurance staff	MOUD clinical staff	
**Active MOUD patients at project initiation**	6	2	40	8	3	**59**
**Active MOUD patients at project termination**	8	2	42	22	32	**106**

^a^Federally Qualified Health Center

±Meeting HRSA FQHC requirements without receiving funding

Bold values indicates the total number of patients for all five clinics

### Engagement in ECHO-AMPLIFI

All five clinics participated in all nine PACT-MAT ECHO sessions and in all six ECHO-AMPLIFI sessions that occurred contemporaneously with the PACT-MAT ECHO. Clinics were offered up to three additional sessions in the three months after the completion of the PACT-MAT ECHO. Out of the nine ECHO-AMPLIFI sessions available to each clinic, actual participation ranged from six to nine sessions over six to nine months, but participation was not always one session per month (see Table 4). The average duration of project participation by clinics was 186 days (range: 140–222 days). All five clinics joined the initial live video presentation introducing the project and a videoconference midway through at which they each shared information about their OUD program structures and protocols related to some of the ECHO-AMPLIFI performance data measures. Three of the five clinics joined a final videoconference to share observations on project participation and impact on their programs (see Table 4).

**Table 4:** Engagement in the ECHO-AMPLIFI project among the five participating clinics

	Clinic 1	Clinic 2	Clinic 3	Clinic 4	Clinic 5	Total
Monthly sessions attended	6	9	7	8	6	**36**
Data submission events	2	3	1	5	1	**11**
Months of patient data submitted	6	1	1	8	1	**17**
Months of panel data submitted	8	4	0	0	0	**12**
Final video-conference participation	No	No	Yes	Yes	Yes	**(3 of 5)** ^*^
Telephone contacts	4	1	2	0	0	**7**
Email exchanges	19	6	7	10	10	**61**

Bold values indicates the proportion of all clinics that participated

Every clinic collected and tracked performance measure data at least once. Between sessions, the five clinics collectively engaged in 61 email exchanges and seven telephone calls with project staff for data collection support and all clinics engaged in some form of remote support (see Table 4). While none of the clinics submitted performance data measures every month, each clinic submitted performance metric data at least one time (see Table 4). Clinics submitted between one and eight months of performance metric data. Clinics collectively provided 11 data submissions containing 17 months of data; some data submissions contained multiple months of data. Months of patient data submitted (17) compared to total monthly sessions (36) was 47% with a median of 28% (range: 28–100%; see Table 4).

Each of the five clinics completed at least one PDSA cycle based on one or more areas targeted by the performance metrics, despite incomplete data collection. As all five clinics attended at least one session, engaged in at least one remote support exchange between sessions, submitted at least one month of performance metric data, completed at least one QI initiative, and attended at least one videoconference, all clinics were considered engaged.

### Barriers to data collection

During conversations with the study team, clinics noted several barriers to data collection. These barriers ran from a lack of personnel available to collect data, issues with the functionality of the data collection tools, and clinic EHR systems being unable document some performance metrics/best practice (e.g., documenting PDMP queries or naloxone distribution). Three clinics reported uncertainty about interpretation of gaps in EHR data. They all noted that absence of EHR documentation of PDMP queries and naloxone distribution in some patient charts may have indicated lapses in clinical performance or may have indicated failure to document actions that did occur. Two of these three clinics responded by developing standardized methods of documenting PDMP queries and naloxone distribution within their EHRs.

### Quality improvement initiatives

Despite barriers with performance data collection, each of the clinics was able to identify at least one QI initiative to work on, and at least two identified their first initiative after a review of performance metrics in the introductory session with the ECHO-AMPLIFI team. One clinic added an ICD-10 diagnostic worksheet to their EHR to promote alignment with best practice [46] and expanded on the project retention measure to include details related to gaps in MOUD and reasons for patients discontinuing treatment to inform clinic follow-up efforts with patients. The clinic reported intent to continue tracking retention, provision of naloxone, PDMP queries, and drug testing for patients receiving buprenorphine beyond the conclusion of the project. Another clinic developed an entry-to-care workflow designating the clinical team member[s] responsible for specific tasks, including screenings and naloxone distribution. They implemented this workflow as their MOUD panel grew from three to 28 patients during the project period. Another clinic responded to preliminary performance data indicating minimal naloxone provision by training 50 staff members in naloxone distribution and developing procedures to document both naloxone distribution and PDMP queries. They also initiated clinic-wide chart reviews for every patient with an OUD diagnosis and developed a plan for clinical response for those not receiving buprenorphine. Another clinic modified their EHR visit template for buprenorphine visits to include dates of PDMP query and naloxone distribution and reported that these changes helped improve the frequency of naloxone distribution and reduce time spent on redundant PDMP queries, though performance data on these measures were not collected. One clinic did not formally launch their OUD program during the project period but prepared to identify internal candidates for buprenorphine by developing a system for SUD screening and identifying current patients with an OUD diagnosis who were not receiving MOUD.

### ECHO-AMPLIFI survey results

Of the 18 staff members from the five participating clinics who received the survey in February 2020, six responded (response rate: 33%). As the survey was anonymous, the distribution of respondents among clinics is unknown. All respondents rated the performance data metrics as quite relevant or extremely relevant to their work. All respondents reported that their confidence in providing buprenorphine services increased and found the practice improvement tools and facilitation useful. 83% of respondents indicated that their experience in the PACT-MAT Project ECHO was enhanced by ECHO-AMPLIFI. Every respondent rated the amount of coaching and data support as the right amount, but one third of the respondents indicated that the total number of months of ECHO-AMPLIFI support was not long enough. Free text responses stated that ECHO-AMPLIFI sessions helped by facilitating conversations and/or thinking about goals and care procedures, and stated these conversations were the most helpful aspects of the project (5 of 6 respondents). However, four (4 of 6) respondents cited data collection as the least helpful aspect of the project. Respondents identified improvements in workflows and tracking naloxone distribution as sustainable changes impacted by ECHO-AMPLIFI (3 of 6) and credited ECHO-AMPLIFI for general improvements in the development of their OUD care with buprenorphine programs (3 of 6) (Table 5).

**Table 5:** Survey results

Survey question	Not at all/a little N (%)	Somewhat N (%)	Quite a bit/extremely N (%)
How relevant were the AMPLIFI data measures to your MAT work?	0 (0%)	0 (0%)	6 (100%)
Did your confidence in providing MAT care increase due to AMPLIFI?	0 (0%)	0 (0%)	6 (100%)
Was your experience in PACT-MAT ECHO enhanced by participating in AMPLIFI?	0 (0%)	1 (17%)	5 (83%)

Survey question	Strongly disagree/disagree N (%)	Neither agree nor disagree N (%)	Agree/strongly agree N (%)
The data collection training and support was useful	0 (0%)	1 (17%)	5 (83%)
The practice improvement tools (PDSA, Process Mapping, 5 Ps) were useful	0 (0%)	0 (0%)	6 (100%)
The onsite practice improvement facilitation was useful	0 (0%)	0 (0%)	6 (100%)
I would recommend AMPLIFI to others	0 (0%)	0 (0%)	6 (100%)

## Discussion

Rising rates of opioid overdose mortality in the United States [1, 2] underscore the importance of expanding access to MOUD [6, 8]. Buprenorphine treatment may offer a combination of accessibility and acceptability most suited to rural areas [7, 9, 16]. To address common barriers to prescribing buprenorphine, developing interventions like Project ECHO to enhance staff training and offer access to experts is critical. This project explored the feasibility of augmenting Project ECHO with the collection of performance data to identify areas for quality improvement at clinics prescribing buprenorphine. Five clinics from mostly rural areas in New Hampshire that were participating in the PACT-MAT Project ECHO engaged in the ECHO-AMPLIFI training sessions, collected at least one month of performance data, and completed at least one QI initiative. Though clinics met expectations for engagement, no clinic engaged in the complete set of activities comprised of attending trainings for nine months, submitting all performance data attending all videoconferences, and all staff responding to the survey. Results support the feasibility of engagement in ECHO-AMPLIFI activities among clinics participating in Project ECHO but did not demonstrate feasibility of engagement in the totality of project activities. Clinic engagement in the ECHO-AMPLIFI training project and survey responses indicate that participating primary care and mental health clinics in rural New Hampshire considered the ECHO-AMPLIFI project instrumental to buprenorphine service delivery improvement and useful in building upon ECHO learning related to OUD.

While they indicated that information gained from performance data collection was useful, clinics encountered multiple barriers to implementation of data collection, including issues with staffing, the functionality of data collection tools, and limitations to documentation within clinic EHR systems. Common barriers to EHR data collection identified through previous research at primary care clinics include challenges configuring EHRs to provide reports, a lack of time and staffing, and difficulty with standardizing documentation of performance measures in EHRs [51–53]. Rurality has been associated with reduced likelihood of generating EHR reports of clinical quality measures [54]. The largely rural Vermont HSS learning collaborative had more data collection reporting but clinics could assign the work to staff supported by the HSS model which linked funding for nurses and behavioral health clinicians to patient volume [38]. This mechanism to increase staff as buprenorphine panels expand may have mitigated the workload barriers identified by New Hampshire clinics. The median reporting rate of ECHO-AMPLIFI performance data was similar to the urban BOOST Collaborative QI coaching project, wherein seventeen health care teams participated in a QI coaching initiative supporting implementation, measurement, and sharing of best practices for MOUD care [35]. That study saw a 35% (range: 0–77%) reporting rate [35]. In settings without mechanisms for increased staff funding, the challenges of EHR configuration and efficient report generation may require regulatory changes to functionality standards required of EHR vendors [54].

That all five clinics engaged with practice facilitation to implement quality improvement initiatives related to buprenorphine services is a finding consistent with utilization of practice facilitation in other healthcare quality improvement projects [55, 56]. The five clinics demonstrated variability in the frequency and number of sessions they attended. Although not directly assessed, this variability suggests that providing flexibility in intensity of activities may be valued by clinic leaders, as has been found previously [57].

Initiatives to standardize documentation of PDMP query and naloxone distribution suggest that OUD care best practices may be enhanced by OUD-specific modifications to the EHR. EHR modification as a QI strategy is not novel, having also been recommended for tobacco treatment [58, 59]. As previous QI research has suggested, it is possible that complete performance datasets are not always essential for the implementation of relevant quality improvement [53]. None of the three clinics that implemented naloxone-specific QI initiatives had collected complete performance data for the measure prior to implementation. Yet two clinics reported that simply tracking naloxone distribution led to improvements in the frequency of providing naloxone to patients, the third designated specific staff to distribute naloxone at specific visits, and a fourth clinic reported that tracking naloxone distribution was important to their buprenorphine prescribers.

Though barriers prevented clinics from collecting complete data sets, clinics demonstrated high motivation to make improvements to better align care processes with best practice of buprenorphine care.

## Limitations

Feasibility was measured through engagement but validated measurement tools of feasibility were not used. This training project was not developed to assess acceptability or impact of data collection and no validated measures of acceptability or appropriateness were employed. Not every buprenorphine prescriber at each participating clinic agreed to attend PACT-MAT ECHO sessions or ECHO-AMPLIFI trainings thus assessment of clinic engagement was not a proxy for assessment of prescriber engagement. While the PACT-MAT ECHO program provided some QI support, it did not include any additional time for clinic staff to engage in data collection. Additionally, ECHO-AMPLIFI did not assess whether tracking performance data resulted in improved quality of care for patients receiving buprenorphine.

Lower attendance at the final videoconference and low survey response rate may have been impacted by the onset of COVID-19, as the videoconference and survey both occurred in February of 2020 in the weeks leading up to the declaration of a national emergency on March 13, 2020 [60]. Finally, this study was conducted with clinics in New Hampshire, a state with a large rural population. While it explores a side of rural care, no comparison to urban medical centers could be made. We do not know whether the challenges clinics faced would be replicated in larger urban settings.

## Conclusions

Findings suggest that implementing performance data-informed QI as a supplement to Project ECHO learning has potential. Consistent with existing literature, clinic resource constraints were found to be a significant barrier to clinic performance monitoring on standardized measures. Yet incomplete performance data collection was shown to be sufficient for some clinically meaningful QI initiatives such as initiating performance tracking of naloxone distribution to increase alignment with best practices of OUD care with buprenorphine. Tracking performance data highlighted gaps in EHR fields at these community health care settings and related QI initiatives indicated that OUD-specific EHR modifications can improve alignment with best practices.

Testing a QI facilitation supplement to a future Project ECHO in more clinics with a smaller set of measures redesigned to minimize burden of data extraction might provide an improved understanding of the potential impact of Project ECHO training enhanced by data-informed QI facilitation. Comparison of clinics participating in Project ECHO augmented by QI facilitation with a control group might better quantify the impact of QI augmentation on adherence to best practices. Such a design might also measure the impact of Project ECHO alone on clinical performance. The finding that complete datasets are not always essential to QI implementation could be further tested through a design measuring the relative impact of QI initiatives based on complete versus partial data sets.

Overall, this study found that implementing performance data-informed QI training as a supplement to a Project ECHO was feasible but had many challenges and limitations. Despite the barriers surrounding data collection itself, the QI efforts made by participating clinics to adhere to best practice for OUD care with buprenorphine indicate that future assessments could provide further information on the utility of performance data in helping busy clinics comply with best practices.

## Data Availability

The Data Use Agreements executed by the five participating clinics limit sharing of performance data submissions and quality improvement status reports to named individuals from the Northeast Node of the Clinical Trials Network and the New Hampshire Citizen’s Health Initiative. These data are not publicly available. Aggregated data with clinic identifiers removed are available from the corresponding author on reasonable request.

## Abbreviations

AMPLIFI: Augmented Medications for Addiction Treatment Practice Learning to Implement Facilitated Quality Improvement
BOOST: Best-practice in Oral Opioid agoniSt Therapy
CTN: National Drug Abuse Treatment Clinical Trials Network
ECHO: Extension for Community Healthcare Outcomes
EHR: Electronic Health Record
FQHC: Federally Qualified Health Center
HSS: Vermont Hub and Spoke System for Opioid Use Disorder Treatment
MOUD: Medications for opioid use disorder
NHCHI: University of New Hampshire Citizens Health Initiative
OUD: Opioid use disorder
PACT-MAT: Partnership for Academic-Clinical Telepractice
PDMP: Prescription Drug Monitoring Program
PDSA: Plan, do, study, act
QI: Quality improvement.
SUD: Substance use disorder
